# Hemolytic Uremic Syndrome: New Developments in Pathogenesis and Treatment

**DOI:** 10.4061/2011/908407

**Published:** 2011-08-17

**Authors:** Olivia Boyer, Patrick Niaudet

**Affiliations:** Service de Néphrologie Pédiatrique, Hôpital Necker-Enfants Malades, 149 rue de Sèvres, 75015 Paris, France

## Abstract

Hemolytic uremic syndrome is defined by the characteristic triad of microangiopathic hemolytic anemia, thrombocytopenia, and acute renal failure. In children, most cases of HUS are caused by Shiga-toxin-producing bacteria, especially Escherichia coli O157:H7. Common vehicles of transmission include ground beef, unpasteurized milk, and municipal or swimming water. Shiga-toxin-associated HUS is a main cause of acute renal failure in young children. Management remains supportive as there is at present no specific therapy to ameliorate the prognosis. Immediate outcome is most often favourable but long-term renal sequelae are frequent due to nephron loss. Atypical HUS represents 5% of cases. In the past 15 years, mutations in complement regulators of the alternative pathway have been identified in almost 60% of cases, leading to excessive complement activation. The disease has a relapsing course and more than half of the patients either die or progress to end-stage renal failure. Recurrence after renal transplantation is frequent.

## 1. Introduction

The hemolytic-uremic syndrome (HUS) is defined by the association of hemolytic anemia (low haptoglobin levels, high lactate deshydrogenase levels, and schistocytes), thrombocytopenia, and acute renal failure [1]. It is a common cause of acute renal failure in patients younger than three years who require acute dialysis. The renal histological lesion is glomerular thrombotic microangiopathy and in more severe cases, arteriolar thrombotic microangiopathy. Increased platelet aggregation and thrombi affect the microcirculation. Thrombotic microangiopathy may affect other organ systems, including the central nervous system [2].

HUS can be divided into two forms, typical and atypical HUS. Typical D^+^ or shiga-toxin associated HUS is the most common form of HUS in children accounting for 90 percent of all cases. It usually occurs after a prodromal episode of diarrhea that is frequently bloody. In the majority of cases, HUS is associated Shiga toxin-producing enterohemorrhagic Escherichia coli (EHEC) or Shigella [3–7] and generally carries a good prognosis [6]. Atypical HUS (aHUS) is a heterogeneous disorder which is responsible for only 10 percent of cases in children [8, 9]. It is also referred to as nondiarrhea-associated HUS, D-HUS, or sporadic HUS. In addition to the usual absence of a diarrheal prodrome, children with aHUS may have an insidious onset, a relapsing course, or a progressive course leading to severe renal dysfunction [1, 8, 10]. Blood pressure is often markedly increased. There is a high risk of recurrence after renal transplantation.

## 2. Shiga-Toxin-(Stx-) Associated HUS

In the majority of cases, Stx-HUS is triggered by strains of EHEC that produce a Shiga toxin [3, 7, 11], but Shigella dysenteriae can also be involved [4, 12]. It usually occurs after a prodromal episode of diarrhea that is frequently bloody in nature. Five to 10 days after onset, HUS develops abruptly [13, 14]. Cases of HUS in children due to Shiga toxin-producing E. coli infections other than colitis (e.g., urinary tract infections) can occur [15]. Although these cases are not associated with diarrhea, the clinical course and pathophysiology are the same as the D+ HUS. 

### 2.1. Epidemiology

Stx-HUS principally affects young children. The annual incidence of the disease in North America and Western Europe is about 2 to 3 per 100,000 children less than five years of age [6, 12]. Stx-HUS occurs most commonly in the summer months [11] and is more frequent in rural versus urban populations [7]. Most cases are sporadic, but outbreaks are often reported due to a common contaminated food or water source. The natural reservoir of EHEC is the digestive tract of healthy cattles Infection in humans occurs following ingestion of contaminated undercooked meat, unpasteurized milk or milk products, water, fruits, or vegetables [16]. Secondary human-to-human transmission is also a major risk factor for HUS [14, 17] and may be a concern in day-care centers or in sibships. 

EHEC causes at least 70 percent of cases of postdiarrheal HUS in the United States, 80 percent of which are caused by E. coli O157:H7, but non-O157 strains are also involved. Although laboratory diagnosis of EHEC infection [18] can be made by stool culture, the bacteria are only present in the stools for a few days and, even if present, may not be detected by culture from stool samples. The rate of stool isolation is substantially higher in the first six days after onset of diarrhea. Direct detection of shigatoxin in stool is another diagnostic tool. Only 10–15% of children with E. coli O157 colitis eventually develop HUS [19] suggesting that, in addition to pathogen factors, host factors also contribute to its development. Shigella dysenteriae type 1-associated HUS occurs in India, Bangladesh, and southern Africa. Although the pathogenesis of disease is similar to that of HUS induced by O157 E. coli infection, the disease is usually more severe with an acute mortality rate of 15 percent and over 40 percent of patients developing chronic renal failure [12]. 

E. coli infection has become a major health care concern. Prevention recommendation should be provided to patients and their parents including meticulous hygiene when cooking and changing diapers, avoidance of undercooked meat and unpasteurized milk products in young children. Cattle vaccination could also help reducing the reservoir of E. coli. Promising results have been obtained in reducing the fecal shedding of E. coli O157:H7 and hide contamination following cattle vaccination [20–22]. But larger epidemiological studies are needed to assess a potential beneficial effect of cattle vaccination for food and environmental safety.

### 2.2. Pathophysiology

Shiga toxin-(Stx-) mediated injury to vascular endothelial cells in the kidney, brain, and other organs underlies the pathogenesis of HUS caused by EHEC. These potent cytotoxins are released in the gut by bacteria, enter the blood stream, and cause endothelial injury through the binding to the globotriaosylceramide (Gb3) receptor on the plasma membrane of target cells [23]. Gb3 is a sphingolipid receptor expressed on endothelial cells, podocytes, and proximal tubular cells in human. Stx binding to Gb3 leads to Stx internalization by receptor-mediated endocytosis and its retrograde transport to the endoplasmic reticulum. This triggers a cascade of signalling events, involving NF-*κ*B activation, which induces apoptosis and the binding of leukocytes to endothelial cells [24]. Stx-activated endothelial cells become thrombogenic by a complex mechanism not yet fully unravelled [24]. It has been demonstrated that Stxs modulate the expression of endothelial cell adhesive molecules such as *β*3-integrin subunit, vitronectin receptor, PECAM-1, and P-selectin on the microvasculature [25] and induce the release of cytokines and chemokines by endothelial cells, implicated in platelet activation [26]. Moreover, upon Stx activation, endothelial Von Willebrandt factor has been shown to undergo conformational changes thereby mediating platelet adhesion on activated endothelial cells [25]. Additionally, Stxs stimulate *in vitro* the release of unusually large Von Willebrandt factor strings and impair their cleavage by the metalloprotease ADAMTS-13 [27]. Altogether, these endothelial cell dysfunctions associated to platelet activation may promote platelet adhesion on glomerular endothelial cells, thrombi formation, and glomerular occlusion in Stx-HUS. An *in vitro* study showed that in addition to directly damaging the kidney, Stxs also activate complement and delay the function of its inhibitor, factor H, on the cell surface, leading to indirect kidney damage [28].

Stxs include two major antigenic forms (Stx1 and Stx2) with different subtypes which differ in potency. Stx2a and Stx2d have been reported as the most potent [29]. Stx type-specific mechanisms of endothelial cell injury have been suggested in cellular models [30]. Bauwens et al. demonstrated that Stx1 causes both necrosis and apoptosis, whereas Stx2 induces almost exclusively apoptosis in human brain microvascular endothelial cell lines and inmacrovascular endothelial cells [30]. Free toxins have never been detected in the blood of HUS patients, but they have been found on the surface of polymorphonuclear leukocytes [31]. Purified Stxs alone are capable of producing HUS with glomerular thrombotic microangiopathy in large animal models such as baboons [32]. Conversely, rodents develop tubular lesions but no glomerular damage after Stx injection. This may be related to Gb3 expression differences among species. Indeed, Gb3 expressed on tubular cells is absent from glomerular endothelial cells and podocytes in mice [23]. Similarly, cattle which are the main vectors of EHEC lack Gb3 expression in their glomeruli. This may partly explain why they do not develop HUS [33]. This led to the hypothesis that different patterns of Gb3 expression with age could explain the predominance of HUS among young children. However, it was shown that Gb3 expression in the kidney and Stx1 binding to kidney structures does not vary with age [34]. Factors other than basal renal Gb3 expression, yet to be identified, account for the age-related incidence of Stx-HUS in EHEC infection. The increased frequency of antibodies to Stx1 and Stx2 suggests that preexisting immunity may play a role in age-specific host resistance to HUS [35].

### 2.3. Clinical Manifestations

Stx-producing E. coli causes a spectrum of outcomes ranging from asymptomatic carriage to diarrhea that may be uncomplicated or bloody to HUS. The diarrhea and associated gastrointestinal complaints in the prodromal phase of Stx-HUS may mimic those of ulcerative colitis, other enteric infections, and appendicitis. HUS is defined by the sudden onset of microangiopathic hemolytic anemia with fragmented erythrocytes named schistocytes and negative Coombs' tests, thrombocytopenia, and acute kidney injury [11]. Despite the thrombocytopenia, there is usually no purpura or active bleeding. Whereas the degree of anemia or thrombocytopenia is unrelated to the severity of renal dysfunction, an increased white blood cell count is associated with a worse prognosis [36]. The hematologic manifestations of Stx HUS completely resolve usually within one to two weeks. 

The renal involvement ranges from hematuria usually microscopic and proteinuria, to severe renal failure and oliguria that occur in one-half of cases. Hypertension is common. Dialysis is instituted to correct metabolic abnormalities when required. The short-term renal prognosis is generally favourable. However, the risk of renal failure 20 years after the recovery of Stx-HUS is not negligible, and renal histology showing a glomerular microangiopathy affecting >50 percent of glomeruli, arterial microangiopathy, and/or cortical necrosis is the best indicator of long-term prognosis [37, 38]. 

Stx-HUS commonly affects other organ systems [36]. Central nervous system involvement occurring in 20–50 percent of children with Stx-HUS is the most threatening complication associated with increased morbidity and mortality [2, 39, 40]. Patients may present with seizures, coma, stroke, hemiparesis, facial palsy, pyramidal or extrapyramidal syndromes, dysphasia, diplopia and cortical blindness [39]. Brain MRI typically reveals bilateral hypersignal on T2-weighted and hyposignal on T1-weighted images of basal ganglia, thalami, and brainstem sometimes extending to the surrounding white matter [41]. Additionally, MRI may display images of high blood pressure complications such as reversible posterior leukoencephalopathy syndrome and cerebral hemorrhage. In a large French multicenter series of 52 children with Stx-HUS and neurological involvement, nine patients died, 17 had mild to severe sequelae, and 26 fully recovered [2]. 

The whole digestive tract can also be involved. The more serious manifestations include severe hemorrhagic colitis, bowel necrosis and perforation, rectal prolapse, peritonitis, and intussusception [42]. Cardiac ischemia and dysfunction may occur. Transient and rarely permanent diabetes mellitus may occur [42]. Hepatomegaly and/or increased serum transaminases are frequently observed. The mortality rate has dropped to about 5%, usually due to neurologic or cardiac involvement [43]. Persistent oliguria (>5 days of anuria and >10 days of oliguria), dehydration, elevated white blood cells > 20,000 per mm^3^, and hematocrit > 23 percent are risk factors for death [43] and long-term complications from HUS [44].

### 2.4. Therapy

Once a patient is infected with EHEC, attempts to prevent progression from the bloody diarrheal phase to the postdiarrheal phase of HUS have been unsuccessful. Antimotility drugs (such as anticholinergic agents and narcotics) do not reduce the progression to HUS due to EHEC infections, but in fact appear to increase the risk of subsequent development of HUS. In patients with EHEC, both retrospective and prospective observational studies report an increased risk of HUS with the administration of antibiotics during the bloody diarrheal phase [45]. In one retrospective review of 278 children with HUS, the use of antimotility drugs (anticholinergic agents and narcotics) was associated with an increased risk of subsequent development of HUS (OR 2.9, 95% CI 1.2 to 7.5) [46]. In another retrospective study of 91 patients, antimotility drugs increased the risk of central nervous system dysfunction (OR 8.5, 95% CI 1.7 to 42.8) [40]. Based upon these data, antibiotics and antimotility drugs should not be given to a child with confirmed or suspected E. coli O157:H7 infection. Increased volume expansion with intravenous isotonic saline during the diarrheal phase has been reported to attenuate but not prevent renal injury [6, 47]. 

Treatment of Stx-HUS includes supportive measures such as management of anemia, significant clinical bleeding, fluid and electrolyte disturbances, hypertension, and other extrarenal complications, together with renal replacement therapy required in 30–60% of cases [48]. Additionally, multiple modalities and/or agents have been utilized that are directed against the underlying or presumed pathogenic mechanisms of Stx-HUS. These include antithrombotic agents, plasma exchange and/or plasma infusion, tissue-type plasminogen activator, and oral Shiga toxin-binding agent. Although none of these agents have been shown to be efficacious, there may be a role for plasma exchange in patients with severe CNS involvement. The remaining agents which have not proven efficient are not recommended. Stx-receptor (Gb3) mimics and Stx-neutralizing (monoclonal) antibodies (STmAb) may be more promising. Although the first-generation Gb3-analog failed to ameliorate the disease course in a randomized placebo-controlled trial [49], advanced Stx receptor analogs and polymers, and peptide-based intracellular toxin inhibitors have been developed and could have more beneficial effects in the future [50–52]. STmAb injection 24 h after oro-gastric inoculation of Stx2-producing bacteria prolonged the survival of toxin-challenged mice [53]. This provided the rationale for the use of STmAb products for Phase 1 studies in patients in which a satisfactory tolerability was demonstrated [54]. Randomized controlled trials are needed to confirm the potential role of STmAb in patients with Stx-HUS. Eculizumab looks promising in severe cases with neurological involvement refractory to plasma therapy [55]. Finally, treatments targeting downstream events in the inflammatory or proapoptotic cascade induced by Stx could be a good option, yet to be developed.

## 3. Atypical HUS

An increasing number of genetic causes of aHUS have been described [1]. These include aHUS associated with mutation of genes encoding for regulators of the alternative complement pathway, Von Willebrand factor cleaving protease (ADAMTS13) deficiency, and intracellular defects of vitamin B12 metabolism. There are also cases of unknown etiology that are associated with an autosomal recessive and autosomal dominant inheritance. 

### 3.1. Atypical HUS and Defective Complement Regulation

Mutations in the genes encoding complement proteins including C3, factors H, B, and I, and membrane cofactor protein (MCP) are associated with atypical HUS [56, 57]. It is estimated that approximately 60 percent of cases of aHUS result from mutations in these genes [56]. These mutations result in dysregulation of the complement system that leads to excessive complement activation and in endothelial damage. 

The gene that is affected determines the clinical presentation and outcome [58, 59]. For example, patients with mutations of the gene for factor H (*CFH*) often progress to ESRD within the first year of presentation. By contrast, only few patients with MCP mutations progress to ESRD, although relapse is common [59]. Interestingly, only 50% of individuals with *CFH* or *CFI* or MCP mutation eventually develop aHUS, which mean that additional risk factors are involved in the development of the disease. Combined mutations of the genes encoding complement proteins are observed in 5% of patients. The association of *CFH* and MCP haplotypes with aHUS has been reported [60]. 

#### 3.1.1. Factor H Mutations

The association between aHUS and mutations in *CFH* was first described by Warwicker et al. [61]. It was subsequently found that it is the most frequent genetic abnormality in patients with aHUS, accounting for 25 to 30% of cases [62–65]. Most mutations are heterozygous. Factor H, a serum complement regulatory protease, acts as a cofactor for factor I-mediated inactivation of C3b, competes with factor B for C3b binding, and accelerates C3 convertase decay. More than 70 mutations have been reported. Most mutations affect the short consensus repeats 19 and 20 of the protein. Most of them are missense mutations, which do not affect the levels of factor H and C3 but affect the C-terminal region, which is important for binding to C3b and anionic surfaces [66, 67]. Other mutations are located throughout the gene and are associated with low levels of factor H antigen. Interestingly, renal survival is higher in patients with low factor H levels compared with those with normal levels of factor H. Patients with homozygous mutations have very low levels of factor H antigen, low C3, low factor B, and low CH50. aHUS associated with factor H mutations may present during infancy or early childhood, or in adulthood [62, 63, 68–71]. The disease may be sporadic or clearly associated with a family history of disease. Hemolytic anemia is marked at disease onset and during relapses, with haptoglobin levels remaining low during the course of the disease. Hypertension is frequently severe. Progression to end-stage renal disease is often rapid, and there is a very high risk of recurrence after renal transplantation (75 to 90%) [72, 73].

#### 3.1.2. Mutations in MCP (Membrane Cofactor Protein)

Mutations in the complement regulatory protein MCP are observed in 5 to 10% of aHUS [58, 74–76]. MCP is the cofactor of factor I for the degradation of C3b and C4b. The mutation results either in a reduction of cell surface levels of MCP (most common) or an impaired function of MCP [75]. The disease is characterized by onset in childhood, a favourable renal outcome in most patients, frequent relapses and a low rate of recurrence in the renal allograft.

#### 3.1.3. Factor I Mutations

Factor I is a serine protease which cleaves C3b and C4b in the presence of factor H and MCP. The frequency of CFI mutations in patients with aHUS varies between 3 and 10% [77]. Up to 15 different mutations have been identified [78]. Most patients have heterozygous mutations [79]. They result in either a quantitative defect or a qualitative defect of the protein. HUS often occur in early childhood, and progression to end-stage renal disease is observed in more than 50% of cases [80]. Other genetic anomalies are frequent and may explain a worse outcome in some patients. Recurrence occurs in 45 to 80% of patients, resulting in graft loss.

#### 3.1.4. C3 Mutations

Heterozygous C3 mutations have been identified in patients with aHUS [81]. Fremeaux-Bacchi et al. reported 9 novel mutations in 14 patients with aHUS and persistently low serum C3 levels. Five of the seven C3 mutant proteins showed a reduced binding to MCP, possibly leading to a gain of function relative to complement activation. 

The mean age at presentation was 6.5 years (range 8 months to 40 years). Seven of the 14 patients regained function after their initial presentation and four of these patients had recurrent disease. Among the 14 patients, there have been 12 renal transplantations, five of which have had recurrent disease.

#### 3.1.5. Factor B Mutations

Mutations of complement factor B that either enhance the formation or delay the inactivation of C3bBb convertase have been reported in 1 to 3% of cases of aHUS [82, 83]. Progression to end-stage renal disease occurs in 70% of patients and all four renal transplantation reported failed because of HUS recurrence.

#### 3.1.6. Thrombomodulin Mutations

Thrombomodulin is a cofactor in the initiation of the protein C anticoagulant pathway. In addition, it was shown that thrombomodulin accelerates the inactivation of C3b by factor I. In a study of 152 patients with atypical HUS, seven patients had six different heterozygous mutations of the *THBD* gene, which encodes thrombomodulin [84]. *In vitro* studies demonstrated that these mutations resulted in a decreased capacity to inactivate C3b, which may lead to excessive complement activation.

#### 3.1.7. Antifactor H Autoantibody-Associated aHUS

The presence of autoantibodies against factor H is observed in 6 to 10% of cases, mainly in children, between 9 and 12 years. These antibodies interfere with the binding of factor H to the C3 convertase and are associated with a defective factor H-dependent cell protection [85, 86]. Most patients have a homozygous deletion of *CFHR1* and *CFHR3* genes [87–89]. The clinical course is characterized by a high frequency of relapses. Progression to end-stage renal disease is observed in 20 to 35% of cases [90]. Plasma exchange plus immunosuppressive therapy have been successful measures in some patients with factor H antibodies [91]. Recurrence is observed when the autoantibodies are still present at time of transplantation [92].

#### 3.1.8. Treatment

Intensive plasma exchange (40 to 60 mL/kg) is the first-line therapy for patients during the acute episode of aHUS. The response to plasma treatment appears to vary depending upon the affected complement component [58, 93]. In patients with either factor H or factor I deficiencies, about two-thirds of patients will remit with plasma therapy. In contrast, there has been no difference in remission rates with or without plasma therapy in patients with MCP deficiency. Patients with antifactor H autoantibodies should receive an immunosuppressive treatment in addition to plasma exchanges.

Eculizumab is a monoclonal antibody which binds to C5 and prevents the generation of C5a and the formation of the membrane attack complex. Eculizumab has been used in patients with aHUS on native kidney or recurrence of aHUS after transplantation with very encouraging results [94–97]. A prolonged therapy is more effective than a single dose. The delay between two injections should not exceed 14 days. The duration of therapy is still a matter of debate. A multicenter trial in children to establish the efficacy and safety of eculizumab in aHUS is ongoing.

Patients with mutations in genes for factor H, factor I, or C3 who fail to respond to plasma therapy and/or have recurrent disease are likely to progress to ESRD [98]. It is unclear whether renal transplantation is an appropriate intervention in these patients, because recurrence of disease occurs in 50 percent of the transplanted kidney, and graft failure occurs in 90 percent of those with recurrent disease [72, 99]. Genotyping for complement protein genes should be performed in patients who have atypical HUS and end-stage renal failure and are being considered for renal transplantation. Indeed, the risk of recurrence is dependent on the type of mutation; such genotyping is particularly important when living-related renal transplantation is being considered, so that cases in which *de novo *HUS occurs in the donor can be avoided. Most clinicians agree that living donor renal transplantation is contraindicated in patients with atypical HUS because of the high risk of disease recurrence after transplantation [1].

In 2007, a consensus conference recommended a combined liver-renal transplantation in aHUS cases in which renal transplantation is extremely unlikely to succeed [100, 101]. This would include patients with factor H or I deficiency who have lost an isolated kidney transplant due to disease recurrence or have a family member with the same gene mutation who lost a kidney transplant due to disease recurrence. The transplantation should be preceded by plasma exchange, and plasma should be infused intraoperatively and continued until liver function is adequate. Eculizumab is another option [94, 96].

#### 3.1.9. Atypical HUS and Von Willebrand Factor Cleaving Protease Deficiency

Von Willebrand factor (VWF), a glycoprotein that carries factor VIII in the circulation, is required for platelet adhesion and platelet aggregation [102]. Large multimers of VWF are more effective than dimers for platelet adhesion and platelet aggregation. The large multimers do not normally circulate because they are cleaved by a specific protease, a metalloprotease synthesized by the liver. This protease is the thirteenth member of the ADAMTS family, or ADAMTS13. 

Many cases of adult thrombotic thrombocytopenic purpura (TTP) are due to deficient activity of the VWF cleaving protease. Protease deficiency may be inherited by autosomal-recessive transmission or may be induced by an acquired autoantibody that inhibits protease activity [103, 104]. A few cases of antibody-induced VWF cleaving protease deficiency have been reported in children aged 1 to 11 years who presented with symptoms of TTP.

Most children with VWF-cleaving protease deficiency related to mutations in the ADAMTS13 gene present at birth with hemolytic anemia and thrombocytopenia. Renal involvement often occurs later in life and has a progressive course. Onset of disease up to five years of age has also been reported. Many develop symptoms of CNS involvement.

#### 3.1.10. Atipical HUS and Defects of Vitamin B12 Metabolism

Vitamin B12 or cobalamin is the coenzyme of methionine-synthase (or methyl-transferase), which transforms homocysteine in methionine. It is also the coenzyme of methylmalonyl-CoA mutase, which is involved in the conversion of methylmalonyl CoA to succinyl-CoA [105]. Children affected with cobalamin C mutations have a functional defect of both methylmalonyl CoA mutase and methionine-synthase. This defect results in methylmalonic acidemia with homocystinuria.

Approximately 25 percent of reported cases also have atypical HUS [105]. The first symptoms, which are nonspecific, occur in the neonatal period and consist of anorexia, vomiting, failure to thrive, hypotonia, seizures, and lethargy [106, 107]. The first symptoms of HUS are observed between the end of the first month and three months of age with severe hemolytic anemia with schistocytes, which is accompanied by macrocytosis, frequent thrombocytopenia, hematuria, proteinuria, renal dysfunction of variable severity, and hypertension.

Amino acid and organic acid chromatography reveal a marked increase of homocysteine and a low level of methionine in the plasma, while urinary excretion of homocysteine and methylmalonic acid is very high. Therapy with hydroxycobalamin should be started as soon as possible [107].

### 3.2. Other Causes of Atypical HUS

HUS may occur during treatments with cytotoxic drugs, such as mitomycin C, bleomycin, or cisplatin [108, 109]. HUS has been described in patients with HIV infection [110, 111]. HUS has been observed in patients with systemic lupus erythematosus [112] as well as in those with a catastrophic antiphospholipid syndrome.

### 3.3. HUS after Renal Transplantation

HUS may be observed following renal transplantation either as a recurrent or a *de novo* disease [113]. More than 50% of patients with aHUS have mutations in genes coding for regulatory factors of the complement system or antibodies against factor H. The risk of recurrence depends on the genetic defect. When the risk of recurrence is high, as in patients with mutations in genes coding for circulating regulators of complement, isolated renal transplantation is contraindicated given the high risk of recurrence, although the use of eculizumab may allow such procedure.

Drug-induced HUS has been observed in transplant patients treated with either cyclosporine or tacrolimus [114, 115]. It usually occurs during the first few months posttransplantation, a time when high doses of calcineurin inhibitors are being administered.
